# Transition Metal Dichalcogenides (TMDC)-Based Nanozymes for Biosensing and Therapeutic Applications

**DOI:** 10.3390/ma15010337

**Published:** 2022-01-04

**Authors:** Dario Presutti, Tarun Agarwal, Atefeh Zarepour, Nehar Celikkin, Sara Hooshmand, Chinmay Nayak, Matineh Ghomi, Ali Zarrabi, Marco Costantini, Birendra Behera, Tapas Kumar Maiti

**Affiliations:** 1 Institute of Physical Chemistry, Polish Academy of Sciences, 01-224 Warsaw, Poland; dpresutti@ichf.edu.pl (D.P.); ncelikkn@ichf.edu.pl (N.C.); 2 Department of Biotechnology, Indian Institute of Technology, Kharagpur 721302, West Bengal, India; tarun3agarwal5@gmail.com; 3 Department of Biomedical Engineering, Faculty of Engineering and Natural Sciences, Istinye University, Istanbul 34396, Turkey; atefeh.zarepour@gmail.com (A.Z.); ali.zarrabi@istinye.edu.tr (A.Z.); 4 Nanotechnology Research and Application Center (SUNUM), Sabanci University, Tuzla, Istanbul 34956, Turkey; s_hooshmand@yahoo.com; 5 Department of Biotechnology and Bioinformatics, Sambalpur University, Sambalpur 768019, Odisha, India; chinunayak015@gmail.com (C.N.); bbehera@suniv.ac.in (B.B.); 6 Chemistry Department, Faculty of Science, Shahid Chamran University of Ahvaz, Ahvaz 61537-53843, Iran; ma_gh@rocketmail.com

**Keywords:** nanozymes, transition metal dichalcogenides, biosensing, anticancer, antimicrobial, cytoprotection

## Abstract

Nanozymes, a type of nanomaterial with enzyme-like properties, are a promising alternative to natural enzymes. In particular, transition metal dichalcogenides (TMDCs, with the general formula MX_2_, where M represents a transition metal and X is a chalcogen element)-based nanozymes have demonstrated exceptional potential in the healthcare and diagnostic sectors. TMDCs have different enzymatic properties due to their unique nano-architecture, high surface area, and semiconducting properties with tunable band gaps. Furthermore, the compatibility of TMDCs with various chemical or physical modification strategies provide a simple and scalable way to engineer and control their enzymatic activity. Here, we discuss recent advances made with TMDC-based nanozymes for biosensing and therapeutic applications. We also discuss their synthesis strategies, various enzymatic properties, current challenges, and the outlook for future developments in this field.

## 1. Introduction

Recent years have witnessed unprecedented advances in scientific research and technological applications, especially in the field of nanotechnology [1,2,3]. A recent example of how nanotechnologies can positively impact our lives is their role in fighting COVID-19 global outbreak, where mRNA vaccines have been made using a nanotechnology-assisted RNA delivery approach. Therefore, it is ever more evident that nanotechnologies are advancing rapidly, and nanomaterials are becoming an important pillar of biomedical research [4].

From a healthcare point of view, there is increasing hopefulness that nanomaterials will bring significant advances both in the treatment and diagnosis of diseases. Such ambitious goals have inspired researchers to develop new nano-platforms capable of performing both operations at the same time. This has led to the establishment of a new multi-disciplinary research domain known as theranostics. Examples of theranostic nanomaterials are iron-based metal oxide magnetic nanocrystals, MXene, black phosphorus (BP), graphene oxide (GO), manganese dioxide (MnO_2_), and palladium (Pd) [5,6]. In addition to those, an emerging class of materials with theranostic potential is represented by nanozymes (NZs).

Nanozymes (NZs)—a class of nanomaterials exhibiting enzyme-like properties and activities—have been tested for a variety of biomedical applications and are promising alternatives to natural enzymes. These abilities are linked to their inherent nanostructures, which mimic natural enzyme active site or charge/electron transfer [7,8,9]. The growing interest in NZs is also justified by the fact that these nanomaterials can be easily synthesized and functionalized at a low cost, and their catalytic activities can be tuned without compromising their stability. Furthermore, when compared to natural enzymes or traditional organic enzymes, NZs have a long half-life and are simple to store/manage. NZs with peroxidase (POD), oxidase (OD), catalase (CAT), or superoxide dismutase (SOD)-like activities are the most studied and frequently used for one or more applications, including biological sensing, molecular detection, environmental management, immunoassays, and theranostic applications [8,10,11].

From the material perspective, zero-dimensional (0D) nanomaterials—based on metal, bimetallic compounds, metal oxides, and metal chalcogenides—were the first NZs to be studied [12]. Despite many advantages, 0D NZs suffer from some limitations, including considerable toxicity, low catalytic activity, and the steric hindrance, which impairs the full enzymatic mimicry. Therefore, researchers have developed new strategies to fabricate two-dimensional (2D) nanomaterials [13]. The two most distinguishing features of 2D nanomaterials are—(i) the lateral dimensions, which are often one or several orders of magnitude larger than their thickness and (ii) the numerous reservoirs and abundant anchoring sites present on their surface to load and deliver therapeutic agents [13]. These materials may exist as nanosheets, nanoribbons, nanoplates, and nanowalls [14]. The primary 2D nanomaterials are transition metal dichalcogenides (TMDCs), transition metal oxides (TMOs), metal carbides/nitrides (MXenes), graphitic carbon nitride (g-C_3_N_4_), hexagonal boron nitride (h-BN), and metal-organic frameworks (MOFs) [13]. TMDCs—with the general formula MX_2_, where M represents a transition metal and X is a chalcogen element—have shown exceptional potential in biomedical applications such as biosensing, tissue engineering, bioimaging, and anticancer therapy [13,15,16]. Their unique nano-architecture, high surface area coupled with their unique semiconducting properties with tunable band gaps, impart them different enzymatic properties. Furthermore, the compatibility of these materials with various chemical or physical modification strategies provides an easily scalable method of engineering and controlling their enzymatic performances.

Based on the recent trends and achievements, in this review, we aim at providing a state-of-the-art overview on TMDCs-based NZs with a particular emphasis on biosensing and therapeutic applications.

## 2. TMDC Nanozyme: Synthesis, Enzymatic Activities, Regulatory Factors

TMDC structure comprises of three layers: a central core composed of transition metal atoms (mostly Mo or W) embedded between the top and bottom layers of chalcogen (S or Se). In particular, they exhibit strong in-plane covalent bonds and weak out-of-plane van der Waals forces.

**Figure 1:**
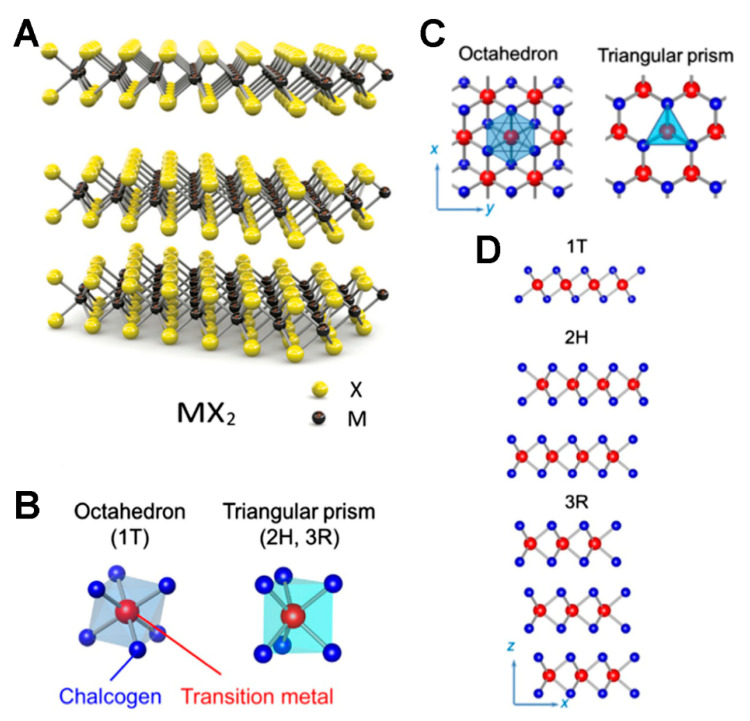
Schematic of (**A**) 3D-structure of TMDCs, (**B**) Octahedron and triangular coordination of TMDCs, and (**C**) top view and (**D**) side view of common forms of octahedron and triangular poly type. Adapted with permission from Ref. [19]. Copyright 2019 Elsevier.

This type of structural feature donates specific properties to TMDCs, for instance, the intercalation of metal atoms between the two chalcogen layers can modify and improve their optical properties. Furthermore, these intercalating agents can boost the superconductivity features of TMDCs, reaching an unprecedented collective electronic phenomenon, impossible to achieve without their use. In other words, the addition of metal atoms induces structural changes that increase the distances between the two chalcogen layers that, in turn, enhance the superconductivity capability. However, this feature could also be achieved via (i) electrostatic or chemical doping or (ii) utilizing pressure [17,18]. TDMCs can show different structural conformations due to the different spherical coordination of the transition metal atoms. Among them, octahedral (1T) and trigonal prismatic (2H and 3R) are the most common polymorph (Figure 1) [19].

Depending on the synthesis method, TMDCs show different colloidal proprieties, producing materials with various levels of water stability. To increase it, TMDCs with low water stability could be treated with biocompatible polymers, using covalent functionalization or physical adsorption methods [20,21].

This ultrathin atomic layer structure confers various other interesting features, including enzymatic properties [15]. Remarkably, it is well established that morphology, shape, size, and surface charge of TMDC NZs could affect their enzyme-like activity. For example, the increase of the size can enhance the rate of electrons transfer, modulating the catalytic activity of TMDC NZs. In the same way, the surface charge can also impact the rate of electrons transfer. For instance, TMDC NZs with negative charges favor the electrons transfer when exposed to substrates that show positive charges on their surface [22]. This section, we would elaborate over the aspects of synthesis, different enzymatic activities, and various factors regulating the activities of TMDC NZs.

### 2.1. Methods for the Synthesis of TMDC NZs

Recently, significant progress has been made in the synthesis of TMDCs nanomaterials using bottom-up (chemical vapor deposition, physical vapor deposition, and hydro/solvothermal methods) and top-down (mechanical exfoliation, solvent exfoliation, and ion-intercalation exfoliation) processes [15]. These methods are based on the use of a diverse set of synthetic precursors and ligands, yielding TMDCs with diverse features, shapes, phases, and applications. However, our discussion here will be limited to the synthesis methods used to prepare TMDC NZs, along with some relevant examples.

#### 2.1.1. Hydro/Solvothermal Method

Hydrothermal or solvothermal synthesis is commonly regarded as the synthesis by chemical reactions of substances in a sealed and heated aqueous solution or organic solvent at high temperatures (100–1000 °C) and associated high pressures (1–100 MPa). Notably, the solvent type and composition can critically affect the geometrical aspects of the synthesized TMDC nanomaterials, highlighting the methods’ plasticity [23]. Hence, the hydro/solvothermal method represent a commonly used strategy for TMDCs synthesis and functionalization. The studies conducted by Fan et al. [24] and Zhan et al. [25] pioneered the route for hydro/solvothermal methods and the production of ultrathin nanoscale TMDCs [26].

To date, the syntheses of POD-like NZs have been conducted through hydrothermal or solvothermal synthesis by different groups using various reducing agents or different reaction media to alter reaction mechanisms and kinetics, attain special conformations, structures, condensed states, and particular morphologies. For instance, sodium salt of molybdenum (Na_2_MoO_4_) in aqueous media with L-cysteine was used to attain MoS_2_ nanosheet with POD-like activity. The reaction took place at 200 °C for 36 h and L-cysteine acted as a sulfide source as well as a reducing agent [27].

Despite the ease of hydrothermal reactions, the variety of organic solvents with different characteristics, such as boiling point and polarity, offers additional room for new and enhanced synthesis techniques. In solvothermal synthesis, the organic solvent not only supplies a reaction medium but also dissolves or partially dissolves the reactants to form a solvent-reactant complex, affecting the chemical reaction rate. For instance, MoSe_2_ particles with POD-like activity have been synthesized through the microwave-assisted solvothermal reaction in N-methyl-2-pyrrolidone in less than an hour. Interestingly, the study evaluated the effect of the reaction temperature on MoSe_2_ phases, i.e., metal 1T phase and semiconductor 2H phase. With a decrease in synthesis temperatures (240, 220, and 180 °C), MoSe_2_ phases transitioned from 2H phase to 1T phase. 2H-MoSe_2_ exhibited good crystallinity and semiconductor properties, whereas 1T structure had a certain degree of disorder and metallic properties. Notably, 1T structures possessed higher POD-like activity than 2H-MoS_2_ [28]. Tungsten disulfide (WS_2_) quantum dots with POD-like activity were synthesized by solvothermal process in dimethylformamide (DMF). Despite the fact that the reaction lasts for 6 h, it occurs at lower temperatures (140 °C) [29].

#### 2.1.2. Chemical Vapor Deposition (CVD) and Physical Vapor Deposition (PVD)

CVD is a chemical process that allows the generation of nanocoating or nanomaterials employing gas/steam reactions. This approach consists in injecting two or more gaseous raw materials into a reaction chamber to enable their interaction and deposit a new material on a molten substrate or a heated solid. It has widely been used to fabricate TMDCs on a large scale, with tunable thickness, such as MoS_2_, MoSe_2_, WS_2_, ReS_2_ nanosheets, and some heterostructures. Precisely, during CVD preparation, transition metal oxides (such as MoO_3_ or WO_3_) are placed with chalcogenide elemental powders (for example, sulfur powder or selenium powder) in a furnace. Exposure to high temperature (700–800 °C) causes the chalcogenide powder to form steam, which then reacts with transition metal oxides to generate thin TMDC films on melted SiO_2_ or sapphire substrates [30,31]. Recently, Gao and colleagues have synthesized a monolayer of WSe_2_ on Au foil, at the millimeter scale, within 30 s, suggesting that the ultrafast method proposed, in which it is possible to modulate the growth time, is useful to control the crystal size if precursors are continuously provided [32]. Remarkably, Appel and colleagues have investigated the biocompatibility of mechanically exfoliated and CVD-grown pristine 2D TMDCs MoS_2_ and WS_2_. The authors did not report any toxic effects on mammalian and bacterial cells. Furthermore, these materials did not alter the level of ROS, suggesting that they can be useful for fabricating medical devices [33]. Chen and co-authors analyzed the biocompatibility of MoS_2_ biosensors synthesized via CVD. Using both in vitro cell assays and in vivo immunological experiments, the research team demonstrated that MoS_2_ is a biocompatible semiconductor. Interestingly, the authors have also investigated the stability of polycrystalline MoS_2_ monolayer (grain size ~200 nm) in the water phase, observing that a complete degradation can be achieved in approximately two months. This study highlighted the capability to integrate TMDC NZs into bioabsorbable and water-soluble electronic platforms to be used in biomedical implants [34].

Apart from CVD, several PVD approaches have also been proposed, including vacuum evaporation, sputtering, arc plasma, ion plating, and molecular beam epitaxy (MBE) for TMDC synthesis. All these PVD methods share common features. In particular, using an intense energy input, it is possible to generate monolayer alloys by direct vaporization of the end TMDCs powder (such as MoS_2_ or MoSe_2_). Successively, at a lower temperature, the vapor can condense on a substrate’s surface under ultra-high vacuum condition (1 × 10^−8^ Torr) or in the presence of ultra-high purity gas. Since TMDCs do not contain dangling bonds and do not require to satisfy lattice matching conditions, the MBE approach is a suitable tool for their synthesis. Therefore, MBE represents the most used PVD production method [15,35].

#### 2.1.3. Exfoliation Method

As a top-down strategy, this method involves stripping TMDC bulks to generate few-layered or monolayered structures. Depending upon the approach opted, this method can further be classified as solvent-based exfoliation and mechanical exfoliation [15,36].

On one hand, the solvent-based exfoliation method entails dispersing TMDC bulk materials in an appropriate solvent before exposing them to ultrasound. The solvent properties, in conjunction with sonication-induced micro/nano-bubbles, separate TMDC monolayers by increasing the distance between the layers and decreasing van der Waals forces. This method offers exfoliation and functionalization of TMDC materials simultaneously. Notably, the selection of an appropriate solvent during the process is critical to prevent the clustering and suspension maintenance of the exfoliated material [37]. Therefore, Hildebrand and Hansen parameters must be considered when choosing a solvent. Mixtures of isopropyl alcohol (IPA)/water, acetone/water, or Tetrahydrofuran/water are frequently preferred. Furthermore, the type of TMDC material also influences the solvent/water ratio selection. According to Shen and colleagues. the proportion of IPA/water for WS_2_ and MoSe_2_ monolayers should be 1:1, while for MoS_2_, it should be 7:3 [37]. As an alternative solvent mixture, ethanol/water mixture was used to attain few-layered MoSe_2_ nanosheets. The commercial MoSe_2_ powder was dispersed in a 45 vol% ethanol/water mixture under ultrasonication (80% amplitude) for 8 h at 10 °C. Indeed, the atomic force microscopy (AFM) and transmission electron microscopy (TEM) analysis clearly indicated an efficient exfoliation of the bulk MoSe_2_ powder [38]. An ethanol/water mixture was also used to obtain WSe_2_ nanosheets; however, ethanol concentration was reduced to 12%. Similar to the previous studies, the exfoliation of the bulk WSe_2_ into WSe_2_ nanosheets was demonstrated with TEM, X-ray powder diffraction (XRD), and AFM analysis. Moreover, regarding its enzymatic activity, WSe_2_ nanosheets show higher POD-like activity than bulk WSe_2_ [39].

Mechanical exfoliation method can also be used, wherein processes such as grinding, ball milling, and scotch-tape causes exfoliation of layers from bulk TMDC crystal [15,36]. In the scotch-tape method, TMDC bulk material is affixed to adhesive tape and folded-unfolded multiple times to allow the material’s thinning. On the other hand, grinding or ball milling methods are fragmented and stripped by friction and collision. Despite their ease of use and low cost, these methods have yet to be used to prepare TMDC-NZs. However, in a recent study, the mechanical grinding process in the presence of ionic liquid (1-butyl-3-methylimidazolium hexafluorophosphate) and chitosan was used to prepare chitosan-functionalized MoSe_2_ nanosheets with POD-like activity [40].

Similarly, ion-intercalation exfoliation (IE) is achieved through the insertion of ion impurities between the layers of TMDC crystal bulks. This increases interlayer space by overcoming van der Waals forces. Due to their high reduction potential and mobility, lithium ion-based intercalants, such as n-butyllithium in hexane, are the most commonly used in this method. The TMDC crystals are hydrolyzed and ultrasonicated to promote efficient intercalation of Li-ions and stacking of the material [24]. As an alternative to the classic IE method, a considerably faster and flexible electrochemical approach can be employed. In this, an electrical voltage is applied between a lithium foil (anode) and a TMDC crystal submerged in an electrolyte solution in the procedure (cathode). As a result, lithium enters between the TMDC layers, and the nanosheets are exfoliated by ultrasonication [41].

### 2.2. Synthesis of TMDC Hybrids with Enzymatic Activity

#### 2.2.1. Doped TMDC NZs

Doping is an effective approach to manipulate the overall performance of TMDC-NZs, resulting from the change in material’s electronic structure. The doping approach often involves the substitution of host atoms with anionic or cationic impurity atoms. In anion substitution, host chalcogenide atoms are replaced with non-metal dopant, whereas in the cationic substitution, impurity atoms replace the host transition metal atoms [36]. It is often observed that the doped TMDC-NZ exhibit better enzymatic activity as compared to the non-doped counterparts as a result of the shifts in the Fermi levels. For instance, a two-step gas expansion and exfoliation strategy was used to obtain nitrogen-doped MoS_2_ (N–MoS_2_) and nitrogen-doped WS_2_ (N–WS_2_) nanosheets. Briefly, the interlayers of bulk MoS_2_ and WS_2_ were expanded with urea molecules in water. Urea which decomposed to NH_3_ during the hydrothermal process and sulfur atoms of TMDCs were partly replaced by N atoms to achieve N doping. The study also revealed that the doping extent was highly dependent on the amount of urea used in the process [42]. Alternatively, plasma treatment, with the merits of low energy consumption and chemical waste-free yield, can also be used for doping. In a recent study, hydrothermally synthesized MoS_2_ nanosheets were exposed to nitrogen plasma to generate N-MoS_2_ NZs that have better POD-like activity and stability than non-doped counterparts [43].

Notably, in relevance to TMDC NZs, cationic doping and its effects on enzymatic performance have still not been fully evaluated and thus, present a scope for further research and development. However, it is well established that tungsten doping on Mo-TMDCs and vice versa are suitable cation substitution processes to handle the optical properties [44]. In the same line, rhenium (Re) and Niobium (Nb) have been used as n- or p-type dopants, respectively, to manipulate the Fermi level of MoS_2_ [45,46].

#### 2.2.2. Functionalized TMDC NZs

Despite TMDCs show good biocompatibility, different modifications (physical or chemical) are often needed to further tune their medical applicability. These modifications often affect colloidal stability, dispersibility, selectivity, and sensitivity of TMDC NZs [11]. In particular, functionalization can change the pH and the temperature at which the TMDC NZs show their activity. Dextran-functionalized MoSe_2_ (dex-MoSe_2_) was synthesized by addition of dextran to the solution containing bulk MoS_2_, followed by ultrasound-mediated exfoliation process. Dextran formed multivalent hydrogen bonding with exfoliated MoS_2_ nanosheets, thereby stabilizing them. These NZs showed POD-like catalytic activity under broad pH conditions, including pH 7.4, which makes it suitable for biological diagnostic applications. Besides, the study also revealed that the catalytic activity of dex-MoSe_2_ was significantly higher than PEG-MoSe_2_ or chitosan-MoSe_2_, produced following the same protocol [37]. Polyvinylpyrrolidone (PVP) was added to the reaction solution for hydrothermal synthesis of ultra-small MoS_2_ nanoparticles to obtain biocompatible POD-like catalytic system. This system exhibited catalytic activity and stability up to 35 °C; however, a marked decrease in activity was observed above 35 °C. Moreover, a good catalytic activity was observed over a broad pH range from 3.5 to 6.5 [47].

Functionalization of TMDC NZs with charged polymers also affects the selectivity to substrates. Positively and negatively charged surfactants, i.e., cetyl trimethyl ammonium bromide (CTAB) and sodium dodecyl sulfate (SDS) respectively, were used to modify solvothermally generated MoS_2_ nanoparticle. The POD-like activity of nanoparticles was obtained to be highly dependent on the surface charge and the highest catalytic activity toward 3,3′,5,5′-tetramethylbenzidine (TMB; positively-charged) was attained with negatively charged SDS–MoS_2_ nanoparticles, primarily due to the high affinity to substrate [48]. In an alternate study, the effect of charge on enzymatic activity and affinity was investigated using positively charged polyethyleneimine (PEI), negatively charged polyacrylic acid (PAA), neutrally charged PVP, and positively/negatively charged cysteine (Cys) to functionalize MoS_2_ nanoflakes (MoS_2_ NFs). The results indicated Cys–MoS_2_ worked well with TMB as well as 2,2′-Azino-bis(3-ethylbenzothiazoline-6-sulfonic acid) diammonium salt (ABTS; negatively-charged) substrates. PAA and PVP modification mildly, while PEI completely blocked the catalytic of NZs [49]. In addition, another study functionalized WS_2_ nanosheets with hemin (iron protoporphyrin), the active center of the heme-protein family, including cytochromes, peroxidases, myoglobin, and hemoglobin. The hemin/WS_2_-NSs has exhibited POD-like catalytic activity at broader pH and temperature range as compared to HRP. Moreover, a higher activity was observed with hemin/WS_2_-NSs than hemin itself or WS_2_ NSs alone [50].

#### 2.2.3. TMDC Nanocomposites with Enzymatic Activity

Besides doping and functionalization, engineering TMDC nanocomposites is also widely studied in order to achieve the optimal enzymatic activity conditions, alter the selectivity and the affinity of TMDC NZs to substrates and creating various nano architectures. For instance, Copper nanowires (Cu NWs) were used as nucleation sites to generate dense, vertically organized, interconnected MoS_2_ NSs. The resulting Cu NW-MoS_2_ NSs composite exhibited a rough surface, allowing better bacterial adhesion and improved POD-like activity compared to both bare Cu NWs and MoS_2_ NSs [51]. Likewise, Wang and colleagues developed MoS_2_/rGO vertical heterostructures with numerous cracks on crystal structure at the basal surface. The increased area of catalytic sites increased the probability of active edge sites exposure and rough surface for bacteria capture, thus improving the antibacterial performance [52]. As an alternative system, 2D/2D heterojunction of MoS_2_ with g-C_3_N_4_ imparted synergistic effects on POD-like activity by efficiently accelerating the electron transport compared to pure g-C_3_N_4_ nanosheets and MoS_2_ NSs [53]. Other than rGO and C_3_N_4_ nanosheets, implementing AuNPs on MoS_2_ quantum dots (AuNPs@MoS_2_-QDs) enhanced and stabilized POD-like activity, which was comparable to HRP. Higher Fermi level and the involvement of excess electrons in the conduction band of AuNPs resulted in an easy electron transfer, leading higher catalytic activity of composites [54]. Other than that, algae-like polypyrrole (Ppy)@MoS_2_ [55] and flower-like MoS_2_@MgFe_2_O_4_ nano-constructs [56] were also reported with enhanced catalytic activity than their parent counterparts.

New frontiers of TMDC-NZs and biocatalysis are the Single-atom (SA) NZs with isolated active metal centers can be anchored on solid supports. Wang and colleagues prepared SA Co–MoS_2_ via assembly of Co nanodiscs on MoS_2_ nanosheets with relatively higher POD activity as compared to only MoS_2_ NZ. Interestingly, the authors reported that occurrence of two different mechanisms in the case of nanocomposite that synergistically elevated enzymatic performance. In particular, SA Co reaction center favored electron transfer mechanism, while MoS_2_ followed Fenton-like mechanism [57]. In the near future, more such SA TMDC NZs systems are certainly expected to be investigated.

### 2.3. Different Enzymatic Activities and Factors Regulating Them

**Figure 2:**
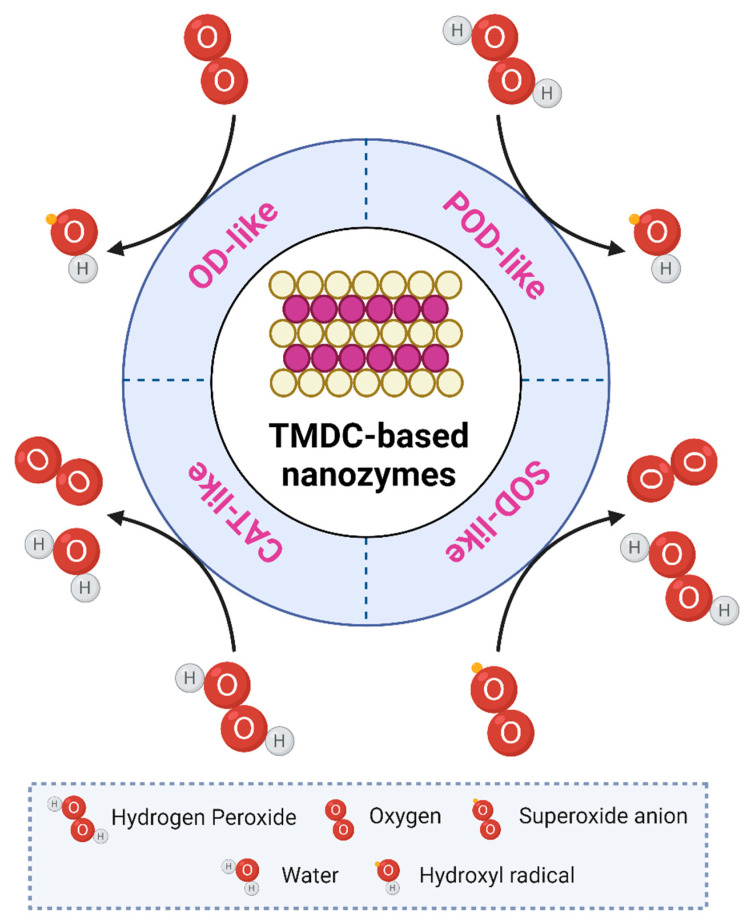
Different enzymatic activities and their mechanisms followed by TMDC-based NZs.

To date, various nanomaterials, including TMDCs, have been reported to display outstanding catalytic activity towards a specific substrate, often following a Michaelis–Menten catalytic kinetic profile. In terms of enzymology, it is proposed that one NZs-Unit represents the quantity of NZs required to catalyze 1 μmol of substrate per minute. Based on the type of catalytic reaction mimicked, NZs can be categorized into several subtypes, including POD-like, OD-like, SOD-like, and CAT-like [13] (Figure 2).

POD activity of TMDC NZs represents the catalysis of peroxides (like hydrogen peroxide (H_2_O_2_)) and results into the oxidation of the substrates, mainly via the production of reactive hydroxyl radical (^●^OH). TMDC NZs with OD activity, in contrast, utilizes oxygen (O_2_) as a substrate to generate ^●^OH, thereby avoiding the usage of unstable and potentially damaging H_2_O_2_. Biosensing (through the utilization of different chromogenic substrates such as TMB, ABTS, and O-phenylenediamine dihydrochloride (OPD) as well as antibacterial and anticancer therapies can all benefit from TMDC NZs with POD/OD-like activity (via oxidative damage to cellular components) [38,58,59]. SOD and CAT mimicking TMDC NZs, on the other hand, have antioxidant properties and play an important role in ROS management. In particular, SOD activity entails disproportionation of ^●^O_2_^−^ into O_2_ and H_2_O_2_, whereas dismutation of H_2_O_2_ into O_2_ and H_2_O occurs in the case of CAT activity. As a result, these TMDC NZs could be used to treat inflammatory diseases like osteoarthritis and neurodegenerative disorders [13].

The sensitivity of NZs determines their potential applicability. The number of active sites/centers and the conductivity of TMDCs, in particular, have an impact on their enzymatic activity. Researchers have made significant efforts to improve the catalytic performance of TMDC NZs. So far, the production of TMDC NZs with, paradoxically, structural defects to create extra edge sites and treatment with different functional groups—cooperatively anchored on their surface—are the most popular ways for promoting enzyme-mimicking activity. Apart from that, other extrinsic factors, such as pH, temperature, exposure to light, and the presence/absence of a particular component (like certain metal ions), can also modulate their performance. For instance, the relative peroxidase activity of WS_2_ quantum dots reduced by 40% from pH 2.2 to pH 5–7, but increased from 20 to 100% when the temperature was raised from 25 °C to 65 °C (via exposure to 808 nm NIR laser irradiation) [60]. In another work, MoS_2_ nanosheets were found to have good POD activity over a wide pH range of 2–7.5, but decreased catalytic activity at temperatures >40 °C [61]. Chitosan modified MoSe_2_ showed optimal POD activity at pH 3.5 and 55 °C temperature; higher pH or lower temperatures were linked with lower activity [28]. The presence of Fe^2+^ ion increased POD activity of WS_2_ NZs [62], whereas the presence of Pb^2+^ ion hindered it [63].

## 3. TMDC Nanozymes: Application Perspective

**Figure 3:**
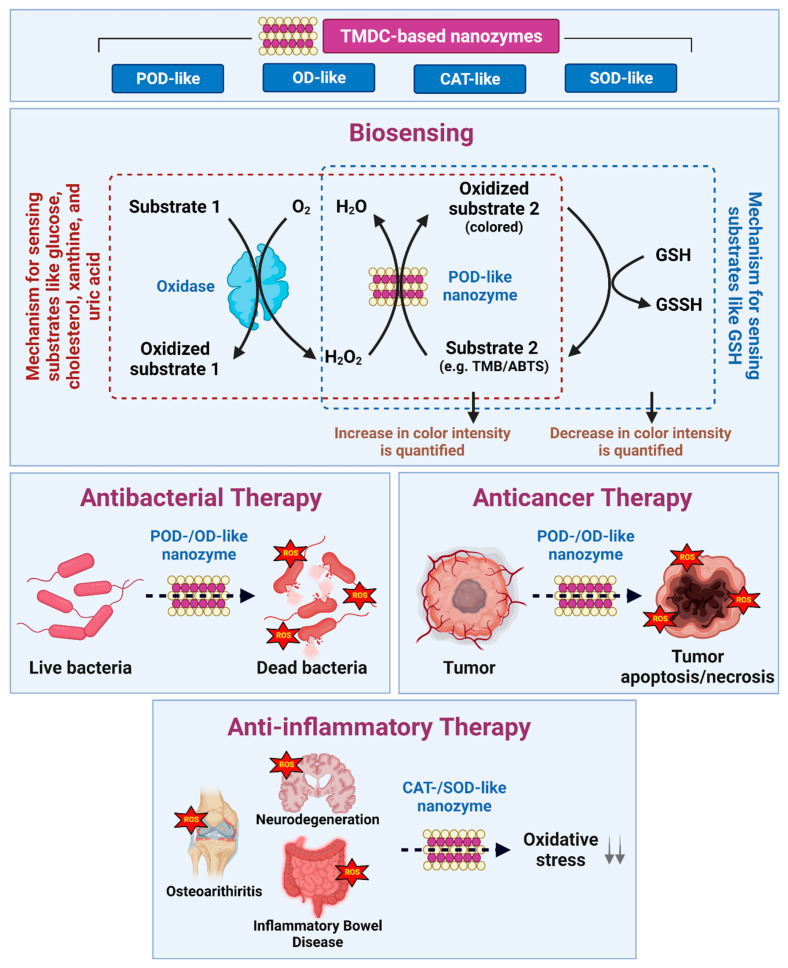
Summary of TMDC-based NZs, their nanozymatic activities, and biomedical applications—biosensing, antibacterial, anticancer, and anti-inflammatory therapy.

In this section, applications of TMDC NZs in different fields—starting from biosensing to different treatment fields like antibacterial, anti-inflammation activity and cancer therapy—are discussed in more details. Figure 3 summarizes the mechanism of action of TMDC NZs to exert various therapeutic and diagnostic effects.

### 3.1. Biosensing Applications

A biosensor is an analytical system that can detect a specific biological analyte and translate presence and/or concentration information into analytical data, such as electrical, optical, and thermal signals, using a simple, low-cost, and time-effective operation [13,64,65]. With the advent of nanotechnology, NZ biosensors, including TMDC-based, have witnessed enormous applicability in biomedical domain, particularly diagnostics, due to their intrinsic enzymatic capabilities [13]. To date, TMDC NZs have been used to detect a variety of biochemical analytes, including tiny biomolecules (such as glucose, cholesterol, glutathione (GSH), and cysteine) as well as macromolecules (e.g., proteins).

TMDC NZ-based biosensing strategies primarily take advantage of their POD-like activity, in which they can oxidize chromogenic substrates (such as TMB, ABTS, and OPD) in the presence of H_2_O_2_ to produce colored products that can be measured colorimetrically [43,55,66]. This NZ-based H_2_O_2_ biosensing is frequently coupled with analyte-specific oxidases such as glucose oxidase (GOx), cholesterol oxidase (ChOx), xanthine oxidase (XOx), and uricase to detect glucose, cholesterol, xanthine, and uric acid, respectively, in biological samples. First, a specific oxidase enzyme metabolizes the bioanalyte in the presence of oxygen to produce a specific acidic product and H_2_O_2_ as a byproduct. This H_2_O_2_ is further sensed colorimetrically by NZs as mentioned above. Notably, within the linear detection range, the intensity of color correlates directly with the amount of bioanalyte present in the samples. The GOx/WS_2_ biosensor system, for example, was used to detect glucose with a linear range of 5–300 μM and a detection limit of 2.9 μM [67]. Similarly, cholesterol was successfully detected at concentrations as low as 15 μM using a ChOx/Au nanoparticle-laden MoS_2_ nanoribbon system [68], whereas uricase/MoS_2_ nanoflakes sensor could detect uric acid within a range of 0.5–100 μM in human serum samples [69].

On the contrary, the detection regimes for cysteine and glutathione (GSH) differ substantially. The ability of these materials to prevent oxidation of colorimetric substrates or revert the oxidized colored product (produced via POD-/OD-like activity of NZs) to its pristine unoxidized form is the basis for their sensing [70]. The color intensity of the reaction mix is inversely proportional to the amount of cysteine or GSH present. Previously, WS_2_ nanomaterial with POD-like activity was used to estimate GSH levels as low as 0.061 nM and a linear detection range of 0.1–10 nM. GSH levels in human serum samples could be measured easily and without interference from other substances [70]. Similarly, cysteine was quantified using Hg^2+^ stimulated OD-like activity of MoS_2_ QDs-Ag NPs in the 1–100 μM range [71].

TMDC NZs can also be used to detect biomacromolecules, such as proteins, in a simple and label-free manner. To date, protein biosensing has been approached in a variety of ways. For instance, lipase was found to prevent POD-like activity of MoS_2_, allowing its detection at concentrations as low as 5 nM [72]. Other TMDC NZs-based protein detection strategies utilize nucleic acid aptamer probes due to their target (proteins or other biomolecules) selectivity, chemical stability, and ability to be synthesized in vitro [73]. ssDNA aptamer probe/MoS_2_ nanosheet system was used to detect carcinoembryonic antigen (CEA). In comparison to bare MoS_2_ nanosheets, the POD-like activity of aptamer/MoS_2_ was ~4.3 times higher, enabling greater oxidation of TMB substrate and consequently higher color intensity. However, when the target analyte, CEA, is present, the attached aptamer probe releases from the MoS_2_ nanosheet’s surface and binds with the protein, showing a reduced TMB oxidation. This drop in color intensity can be measured and is inversely proportional to the CEA concentration. Using this method, CEA could be detected in a linear range of 50–1000 ng/mL with the detection limit of 50 ng/mL [74]. Aptamer-anchored MoS_2_/PtCu nanocomposites with strong OD-like activity were used to detect mucin 1 positive cells with high sensitivity and selectivity. Cells such as MCF-7 and A549, which have mucin 1 overexpression, could be detected even in populations as small as 300 cells. The use of NZs with OD-like activity, as in this case, is often advantageous because it surpasses the use of cytotoxic H_2_O_2_, thus improving the biocompatibility and allowing the biosensor to be used in conjunction with living cells [58]. Besides, protein-specific antibodies [75] or antibody/aptamer probes [76] were also physically/chemically conjugated onto TMDC NZs to detect *Salmonella typhimurium*-specific surface proteins and human epididymis-specific protein 4 (HE4) proteins, respectively.

Table 1 summarizes some of the recent TMDC-based NZs that have been used for molecular and macromolecular biosensing so far.

### 3.2. Therapeutics

#### 3.2.1. Antibacterial Activity

The annual increase in the cases of bacterial infections is one of the most challenging aspects of the global public health and safety [84]. In particular, an alarming rise in bacterial drug resistance—as a result of uncontrolled use of antibiotics—has heightened concerns in this context [85]. Significant efforts are being made to overcome this challenge through the development of novel antibacterial agents with greater efficacy and specificity than conventional antibiotics. Recently, a new type of antibacterial therapy with broadband antimicrobial capability, known as nanozyme-mediated antibacterial therapy (NABT), has been introduced. It entails the use of NZs, including TMDC-based, with POD-/OD-like enzymatic activities to regenerate ROS, which then exerts antibacterial effects via oxidation of the bacterial membrane’s polysaccharides, proteins, and lipids [52].

The antibacterial activity of TMDC NZs, along with their relatively biocompatible nature, may be advantageous during the wound healing process. Bacterial infections have already been shown to delay healing by increasing inflammatory responses at the wound site [86]. As a result, using appropriate antibacterial agents could help to restore and balance the accurate healing microenvironment and avoid any delays. MoSe_2_ nanosheets/carboxyl-modified silk fibroin based wound dressing exerted considerable antibacterial effects on *Escherichia coli* and *Bacillus subtilis* due to their POD-like activity. The studies were conducted both in vitro and in vivo in *E. coli*-infected full-skin defect mice model in the presence of low amounts of H_2_O_2_ [87]. Another study used lysozyme, an enzyme capable of hydrolyzing bacterial cell wall peptidoglycan, as an exfoliating agent to generate MoS_2_ nanosheets. These nanomaterials demonstrated enhanced antibacterial activity against ampicillin-resistant *E. coli* and *B. subtilis*, which was attributed synergistically to the antibacterial activity of lysozyme and the POD-like activity of MoS_2_ nanosheets [88].

Another intriguing study was conducted by Niu and his colleagues, who used a combination of citraconic anhydride-modified polyethyleneimine (PEI)-MoS_2_ nanosheets and a photoacid generator molecule, 2-nitrobenzaldehyde (2-NBA). When 2-NBA was exposed to 365 nm light, the pH of the solution decreased, which activated the POD-like activity of the NZs to produce ROS and impart antibacterial effects. Furthermore, the irradiation time changed the charge of the nanomaterial from negative to positive, thanks to the photoreactive characteristics of citraconic anhydride, allowing Gram selectivity for the developed antimicrobial system [89].

Multimodal therapy is usually considered to be more efficient and effective at imparting antibacterial effects. NZs were combined with photothermal and chemotherapy in a study by encapsulating WS_2_ quantum dots (WS_2_ QDs) and vancomycin in a thermal-sensitive liposome. The use of WS_2_ QDs benefited in two ways: (i) their POD-like activity allowed the generation of ROS and (ii) their photothermal property resulted in heat generation (via 808 nm NIR laser irradiation), causing liposomal rupturing at the targeted site, resulting in a reduction in drug doses required. This anti-biofilm agent demonstrated excellent anti-biofilm activity, eradicating both *E. coli* and Mu50 (vancomycin-intermediate *Staphylococcus aureus* strain) both in vitro and in vivo [60]. Owing to POD-like activity and photothermal properties, PEG-functionalized MoS_2_ nanoflowers imparted an efficient antimicrobial effect and improved wound healing rate in ampicillin-resistant *E. coli*-infected full-skin defect mice models [90]. Another study used mesoporous ruthenium nanoparticle that was loaded and capped with ascorbic acid prodrug and hyaluronic acid, respectively. Ciprofloxacin-coated MoS_2_ nanosheets were further bound to the outer surface of the nanocomposite. Post-administration, hyaluronidase enzyme (produced by bacteria) would reduce the hyaluronic acid capping degradation and release of ascorbic acid and MoS_2_ at the infected wound site. Ascorbic acid/MoS_2_-mediated reactive radical generation, and ruthenium nanoparticles-mediated photothermal therapy, could synergistically eliminate multidrug-resistant bacterial strains in vitro. Furthermore, this therapeutic agent demonstrated promising efficacy in *S. aureus*-infected mice models (tested for biofilm dispersion inhibition) and *S. aureus* and *Pseudomonas aeruginosa* infected mice models (tested for wound healing). On the other hand, Ciprofloxacin loading did not affect the antibacterial potency of these nanocomposites [91].

#### 3.2.2. Cancer Therapy

Cancer is one of the leading causes of death due to its late diagnosis and insufficient effects of currently available treatments (e.g., chemotherapy, radiation therapy, and surgical treatment) [92]. NZs, including those based on TMDC, have recently gained prominence in cancer treatment. NZ-mediated cancer therapy, like antibacterial systems, uses POD-/OD-like activities to generate ROS and cause cancer cells to die [93].

NZ-mediated cancer therapy is often limited by the lower availability of intra-tumoral H_2_O_2_. To address this challenge, recently, MoSe_2_/CoSe_2_@PEG nanosheets were synthesized. Using dissolved O_2_ and photoexcited electrons, this system was able to produce H_2_O_2_ via a sequential single-electron transfer mechanism. Furthermore, this NZ system showed potent dual POD- and CAT-like activities, which ensured efficient generation of ^●^OH and O_2_, respectively. ^●^OH caused mitochondrial damage, whereas O_2_ alleviated hypoxia and served as a source of H_2_O_2_. The anticancer effects were amplified by the nanomaterial’s excellent photothermal characteristics, as well as redox disruptions (through intracellular GSH reduction). Besides, biodegradability and urinal/fecal elimination (within two weeks post-administration) are other notable features of this therapeutic system [94].

In another study, a glucose-responsive, H_2_O_2_ self-supplying nano-catalytic reactor was developed by self-assembly of GOx, tirapazamine (TPZ) and chitosan on the surface of MoS_2_ nanosheets. The catalytic mechanisms involved in the cascade are as follows: (i) catalysis of intra-tumoral glucose by GOx (in the presence of O_2_) to produce H_2_O_2_ and lower the pH; (ii) utilization of H_2_O_2_ by POD-like activity of MoS_2_ nanosheets to produce ROS—to damage the cancer cells. Meanwhile, depletion of O_2_ would activate TPZ, whereas MoS_2_ could utilize GSH to disturb cellular redox balance, further amplifying the anticancer effects. This therapeutic agent demonstrated potent anticancer effects on A549 cells in vitro and A549 tumor-bearing mice models in vivo. In contrast, even at concentrations as high as 100 g/mL, no cytotoxicity was observed in normal human umbilical vein endothelial cells (HUVEC). Furthermore, under in vivo conditions, these nanomaterials did not accumulate in normal organs, but instead degraded and were cleared out of the body, indicating minimal toxicity to normal tissues [95].

Another significant challenge in the field of nanomedicine is the development of advanced theranostic platforms with both therapeutic and diagnostic capabilities. In this regard, 3D porous MoS_2_ nanoflowers were synthesized, then loaded with doxorubicin and coated with PEG-PEI (conjugated with LIM Kinase 2 protein (LMP) nucleolar translocation signal peptide). LMP peptide improved the nanomaterials’ nuclear targetability in cancer cells. Thus, these materials were able to specifically target the cancer cells and could exert potent anticancer effects both in vitro (4T1 cells) and in vivo (4T1 tumor bearing mice model) through pH-responsive/NIR-enhanced doxorubicin delivery into the tumor cells, NIR-induced photothermal effects along with ROS generation due to POD-like activity of MoS_2_ nanoflowers. Furthermore, the excellent photoacoustic properties of these materials allowed for real-time tracking post-intravenous in the 4T1 tumor bearing mice models [96]. A smart hybrid NZ based on MoS_2_-coated bipyramidal gold nanostructure was developed for anticancer therapy and two-photon bioimaging. This hybrid nanomaterial produced considerable ROS due to the POD-like activity of MoS_2_, which was augmented further by irradiation with 808 nm NIR laser due to localized plasmonic effects. Such synergistic ROS generation exerted significant anticancer effects in HeLa cells, as confirmed by two-photon luminescence imaging [97].

#### 3.2.3. Anti-Inflammatory Effect

Apart from the applications listed above, TMDC NZs, particularly those with CAT/SOD-like activity, have also been used as antioxidant materials to provide cytoprotective effects and treat inflammatory diseases/conditions such as osteoarthritis and neurodegeneration [98,99,100]. For example, MoS_2_ nanosheets with CAT/SOD-like activity were synthesized those were able to quench and reduce the levels of free radicals like nitric oxide (^●^NO), ^●^OH, and nitrogen-centered free radicals (^●^DPPH). Furthermore, treating H_2_O_2_-exposed A549 cells with these nanomaterials dramatically reduced oxidative stress [99]. Fullerene-like MoS_2_ (F-MoS_2_) is another interesting TMDC-NZs with CAT-/SOD-like activities under physiological settings that appropriate it for using for the non-surgical treatment of osteoarthritis. F-MoS_2_ was able to catalyze ^●^O_2_^–^ into H_2_O_2_ and then produce water and O_2_. Interestingly F-MoS_2_ was used to protect HUVEC cells from oxidative stress induced by H_2_O_2_. Besides, F-MoS_2_, when coupled with hyaluronic acid (HA), could reduce the excess of ROS and prevent the depolymerization of HA in artificial synovial fluid [100]. TMDC NZ with CAT-/SOD-like activity was used to mitigate the pathology of Alzheimer’s disease by targeting neuronal mitochondria with (3-carboxypropyl)triphenyl-phosphonium bromide-conjugated 1,2-distearoyl-*sn*-glycero-3-phosphoethanolamine-*N*-[amino(PEG)-2000]-functionalized MoS_2_ QDs. When tested in vitro in murine-derived microglia BV2 cells, this nano-formulation dramatically decreased oxidative stress, downregulated pro-inflammatory cytokines, and elevated anti-inflammatory cytokines. Furthermore, in vitro (in BV2 cells) and in vivo (in an Alzheimer’s disease mouse model) tests revealed that these nanomaterials were able to reduce amyloid-beta (Aβ) aggregation-mediated neurotoxicity and eliminate Aβ aggregates. These were attributed to switching microglial polarization from pro-inflammatory M1 to anti-inflammatory M2, presenting a novel pathway to mitigate Alzheimer’s disease pathology [101].

Table 2 listed some of the other studied which were done on the therapeutic application of TMDC NZs.

## 4. Conclusions and Outlook

As of today, NZs have presented themselves as a superior alternative to natural enzymes in various sectors, including industrial, environmental, healthcare, and diagnostics. This review highlights the current advancements made with TMDC NZs. As described in the text, nano-architectural features, high surface area, semiconducting properties with tunable band gaps, chemical/physical modifications, and environmental factors represent key factors regulating the intrinsic enzymatic properties of TMDCs and ensured their biomedical applicability. To date, TMDC NZs with POD-/OD-/CAT-/SOD-mimicking activities have been reported for applications such as biosensing, antibacterial, anticancer, and anti-inflammatory activities. However, to truly move forward, on one hand, we need to work upon certain aspects, including their rational design, microenvironment descriptions, expansion from single to multi-activity mimics, incorporating multi-functionality, and addressing biological effects [103], while on the other hand, new TMDC NZs should be investigated. Controlled synthesis of TMDC NZs is essential to achieve a desirable colloidal stability, uniform size, high yield and enzymatic performance [104]. Furthermore, a limited understanding of the optimal structural features and associated catalytic mechanisms, it becomes quite tricky to predict the selectivity and activity of TMDC NZs. In this regard, appropriate theoretical and experimental models should be established to better understand their structure-function relationship, thereby rationalizing the design of NZs for a specific biomedical application [13,105]. Besides, speculating their biocompatibility, biodistribution, biodegradation, metabolism, short-term and long-term toxicity, and immunogenicity is highly desirable for biological applications. In particular, the functionalization of TMDCs is an attractive approach to modulate these aspects. Moreover, integrating stimuli-responsive features and multi-functional capabilities within these materials could impart better controllability over their performance and reduce undesirable effects (particularly when targeting in vivo therapeutic applications).

In conclusion, despite significant progress achieved in the TMDC NZs field, several aspects still need to be appropriately defined, and the development of novel TMDC NZs could be helpful in facing these issues. Nowadays, the synthesis of uniform structures, scalability, and the reduction of the synthesis costs are the main ongoing challenges. Additionally, it is essential to underline that TMDC NZs are still in the early stage of their use compared to other nanomaterials, such as spherical nanomaterials. Therefore, further in vitro and in vivo pre-clinical studies are needed to thoroughly investigate their biocompatibility and potential side effects. In this context, approaches based on bioinformatics tools coupled with machine learning and artificial intelligence could predict novel TMDC NZs with high enzymatic performance.

## Figures and Tables

**Table 1:** TMDC NZs for biosensing applications.

Analyte Detected	Nanozyme System	Activity	Assisting Enzyme	Detection Type	Substrate Employed	Linear Range	Detection Limit	Stability	Biological Samples	Ref.
H_2_O_2_	MoS_2_	POD-like		Colorimetric	TMB	0.125–1.75 μM	0.08 μM		Lake water	[27]
H_2_O_2_	N-Doped MoS_2_	POD-like		Colorimetric	TMB			6 months		[43]
H_2_O_2_	Au NRs-anchored MoS_2_/C	POD-like		Colorimetric	TMB	10–200 μM	1.82 μM		Cancer cells	[66]
H_2_O_2_	MoS_2_/Ppy	POD-like		Colorimetric	TMB	50–2000 μM	45 μM			[55]
Glucose	MoS_2_	POD-like	GOx	Colorimetric	TMB	5–150 μM	1.2 μM		Human serum	[61]
Glucose	MoS_2_ QDs	POD-like	GOx	Fluorometric		10–1500 μM	5.16 μM		Fetal bovine serum	[77]
Glucose	PTCA-MoS2	POD-like	GOx	Colorimetric	TMB	20–800 µM	18.3 μM	2 months (at 4 °C)	Human serum	[78]
Glucose	MoS_2_-MIL-101(Fe)	POD-like	GOx	Colorimetric	TMB	0.01−15 μM	0.01 μM	1 month	Human serum	[79]
Glucose	MoS_2_@MgFe_2_O_4_	POD-like	GOx	Colorimetric	TMB, ABTS	5–200 μM	2 μM	1 month	Human serum	[56]
Glucose	Cysteine- MoS_2_ NF	POD-like	GOx	Colorimetric	ABTS	50–1000 μM	33.51 μM		Human serum	[49]
Glucose	Dextran-MoSe_2_	POD-like	GOx	Colorimetric	TMB	40–400 µM	28 µM	10 days	Human serum	[37]
Glucose	Chitosan-MoSe_2_	POD-like	GOx	Colorimetric	TMB	5–60 µM	0.71 μM	>1 month	Human serum	[28]
Glucose	SDS–MoS_2_	POD-like	GOx	Colorimetric	TMB	5–500 μM	0.57 μM		Human serum	[48]
Glucose	AuNPs@MoS_2_ QD	POD-like	GOx	Colorimetric	TMB	20–400 μM	0.068 μM	12 days	Human serum, tear and saliva	[54]
Glucose	PVP-MoS_2_ NPs	POD-like	GOx	Colorimetric	TMB	1000–10,000 μM	320 μM		Fetal bovine serum	[47]
Glucose	WS_2_	POD-like	GOx	Colorimetric	TMB	5–300 μM	2.9 μM		Human serum	[67]
Glucose	WS_2_ NS + Ag NCs	POD-like	GOx	Chemiluminescence	Sodium bicarbonate	0.03–20 μM	0.0013 μM		Human serum	[80]
Glucose	Hemin-WS_2_	POD-like	GOx	Colorimetric	TMB	5–200 µM	1.5 μM		Human serum	[50]
Glucose	WSe_2_	POD-like	GOx	Colorimetric	TMB	10–60 μM	10 μM			[39]
Glucose	VS_2_	POD-like	GOx	Colorimetric	TMB	5–250 µM	1.5 µM			[81]
Cholesterol	MoS_2_ NS	POD-like	ChOx	Colorimetric	TMB	2–200 μM	0.76 μM		Human serum	[82]
Cholesterol	MoS_2_ nanoribbons–AuNPs	POD-like	ChOx	Colorimetric	TMB	40–1000 μM	15 μM		Human serum	[68]
Cholesterol	Oxidized GSH-modified MoS_2_ NSs	POD-like	ChOx	Colorimetric	TMB	5.36–800 μM	5.36 μM		Mouse serum	[83]
GSH	WS_2_ NSs	POD-like		Colorimetric	TMB	0.1–10 nM	0.061 nm		Human serum	[70]
Uric acid	MoS_2_ NFs	POD-like	Uricase	Colorimetric	TMB	0.5–100 μM	0.3 μM		Human serum	[69]
Xanthine	MoSe_2_	POD-like	XOx	Colorimetric	TMB	10–320 µM	1.964 μM		Human serum	[38]
Cysteine	MoS_2_ QDs-Ag NPs (stimulated by Hg (II) ion)	OD-like		Colorimetric	TMB	1–100 μM	0.82 μM	1 month	Human serum	[71]
CEA	Aptamer/MoS_2_ NSs	POD-like		Colorimetric	TMB	50–1000 ng/mL	50 ng/mL		Human serum	[74]
Lipase	MoS_2_ NPs	POD-like		Colorimetric	TMB	5–200 nM	4.8 nM			[72]
Mucin 1	Aptamer-MoS_2_/PtCu	OD-like	NA	Colorimetric	TMB	NA	300 cells of MCF-7		MCF-7, A549, HEK293, and HepG2	[58]

**Table 2:** TMDC NZs for therapeutic applications.

Applications	TMDCs Material	Activity Mimics	Targeting Molecule (if Any)	Therapeutic Mechanism	Therapeutic Mediators	Light Characteristics (if Involved)	Activity Assessed Against		In Vivo Evaluation	Ref.
							Microbial Cells	Mammalian Cells		
**Disinfection and wound healing**	MoS_2_/rGO	POD-like, OD-like, CAT-like		ROS-mediated	H_2_O_2_	Xenon lamp (100 mW/cm^2^)	Chloramphenicol-resistant *E. coli* and *S. aureus*		*S. aureus*-infected full-skin defect mice models	[52]
	Fe_3_O_4_@MoS_2_-Ag	POD-like		Ag+ ion-mediated toxicity, ROS-mediated, PTT	H_2_O_2_, Ag^+^ ions	NIR (808 nm, 1 W/cm^2^)	*E. coli*			[102]
	citraconic anhydride modified PEI-MoS_2_	POD-like		Disruption of surface charge, ROS-mediated	H_2_O_2_, 2-nitrobenzaldehyde	UV light (365 nm)	*E. coli* and *S. aureus*		*E. coli* and *S. aureus*-infected full-skin defect mice models	[89]
	WS_2_ QDs-Van@lipo	POD-like, OD-like		ROS-mediated, PTT, Chemotherapy	H_2_O_2_, vancomycine	NIR (808 nm, 1 W/cm^2^)	*E. coli* and Mu50 (vancomycin-intermediate *S. aureus* strain)		Mice models with Mu50-infected abscess	[60]
	Cu NW-supported MoS_2_ NS	POD-like		ROS-mediated, PTT	H_2_O_2_	NIR (808 nm, 1 W/cm^2^)	*E. coli* and *S. aureus*		MRSA-infected full-skin defect mice models	[51]
	N-doped MoS_2_, N-doped WS_2_	POD-like		ROS-mediated	H_2_O_2_		Ampicillin resistant *E. coli* and *B. subtilis*		Ampicillin resistant *E. coli*-infected full-skin defect mice models	[42]
	Lysozyme exfoliated MoS_2_ NSs	POD-like		ROS-mediated	H_2_O_2_		Ampicillin-resistant *E. coli* and *B. subtilis*			[88]
	PEG-MoS_2_ NFs	POD-like		ROS-mediated, Photothermal therapy (PTT)	H_2_O_2_	NIR (808 nm, 1 W/cm^2^)	Ampicillin-resistant *E. coli* and *B. subtilis*		Ampicillin resistant *E. coli*-infected full-skin defect mice models	[90]
	CMSF-MoSe_2_ NSs	POD-like		ROS-mediated	H_2_O_2_		*E. coli* and *B. subtilis*		*E. coli*-infected full-skin defect mice models	[87]
**Anticancer therapy**	Glucose responsive, TMZ-loaded chitosan-MoS_2_	POD-like		ROS-mediated, GSH depletion, hypoxia induced TPZ activation	H_2_O_2_ and TPZ			A549 cells	A549 tumor-bearing mice models	[95]
	AuNBPs@MoS_2_	POD-like		ROS-mediated, PTT	H_2_O_2_	NIR laser (808 nm, 2.0 W/cm^2^)		HeLa cells		[97]
	LNP-PEG-PEI coated, Dox loaded MoS_2_ NFs	POD-like	LNP nucleolar translocation signal peptide	ROS-mediated, CT, PTT, PDT	Dox	NIR laser (808 nm, 3.0 W/cm^2^)		4T1 cells	4T1 tumor-bearing mice models	[96]
	MoSe_2_/CoSe_2_@PEG	POD-like, CAT-like		ROS-mediated, GSH depletion, PTT	H_2_O_2_	NIR laser (808 nm, 1.0 W/cm^2^)		HepG2 cells	Tumor-bearing mice models	[94]
**Cytoprotection**	MoS_2_ NS	CAT-like, SOD-like, POD-like		Scavenging oxidative stress species			*E. coli* and *S. aureus*	A549 cells		[99]
**Neurodegeneration**	TPP-MoS_2_ QDs	CAT-like, SOD-like	TPP (mitochondrial targeting)	Scavenging oxidative stress species				BV-2 cells	Amyloid precursor protein/presenilin 1 (APP/PS1) double transgenic mice	[101]
**Osteoarthritis**	Fullerene-like MoS_2_	CAT-like, SOD-like		Scavenging oxidative stress species				HUVECs		[100]

## Data Availability

All data contained within the article.
